# Sexual Behavior and Knowledge among Adolescents with Perinatally Acquired Human Immunodeficiency Virus Infection Compared to HIV-Uninfected Adolescents at an Urban Tertiary Center in New Jersey

**DOI:** 10.1155/2016/7098027

**Published:** 2016-08-10

**Authors:** Ashlesha Kaushik, Carol Pineda, Helen Kest

**Affiliations:** ^1^Department of Pediatrics, St. Joseph's Children's Hospital, 703 Main Street, Paterson, NJ 07503, USA; ^2^Department of Pediatrics, Division of Pediatric Infectious Diseases, St. Joseph's Children's Hospital, 703 Main Street, Paterson, NJ 07503, USA

## Abstract

*Background*. Sexual behaviors and knowledge among PHIV-infected (PHIV^+^) adolescents in comparison with HIV-uninfected youths are not well understood and continue to be studied actively.* Objective*. To compare sexual behavior and sexual knowledge of PHIV^+^ and HIV-uninfected adolescents at an urban, tertiary-care center in New Jersey.* Study Design*. Modified Centers for Disease Control and Prevention (CDC) Youth Risk Behavior Surveillance questionnaire was administered to PHIV^+^ and HIV-uninfected adolescents to assess and compare sexual behavior and knowledge over a 1-year-period.* Results*. Twenty-seven PHIV^+^ and 100 HIV-uninfected adolescents were studied; 59% PHIV^+^ and 52% HIV-uninfected adolescents were sexually active. A significantly higher proportion of PHIV^+^ adolescents compared to HIV-uninfected adolescents reported ≥1 occasion of unprotected penetrative sex (*p* < 0.0001) and reported multiple (>4) sexual partners (*p* = 0.037). Significantly more PHIV^+^ males reported receptive anal intercourse (*p* < 0.001). About 1/3 of adolescents in both groups were unaware that sexual abstinence can prevent HIV transmission and >80% adolescents in both groups did not consider multiple sexual partners a risk factor for HIV transmission. Only 25% PHIV^+^ adolescents reported disclosing their seropositive status to their first sexual partners.* Conclusions*. High risk sexual behaviors were significantly more prevalent among PHIV^+^ youths; however both groups demonstrated considerable gaps in sexual knowledge. There is an urgent need for heightening awareness about risky behaviors, interventions for prevention, and reproductive health promotion among adolescents.

## 1. Introduction

Perinatally acquired HIV infection (PHIV) remains a huge problem worldwide especially in developing countries. According to the WHO (World Health Organization), approximately 7% of the 36.9 million people living with HIV worldwide in 2014 were infected perinatally [1]. Due to HIV prevention and treatment efforts, PHIV transmission has decreased dramatically in the US and the developed world [2, 3]. However, it is still the most common route of HIV infection in children worldwide [1, 4].

With improvements in survival and increased longevity, PHIV-infected (PHIV^+^) adolescents are faced with numerous challenges such as learning about their HIV status, disclosure issues, and HIV transmission risk. They are also confronted with other challenges of the adolescent period such as sexuality, alcohol, and drug experimentation that place them at an even higher risk of disease propagation [5].

Studies have examined risky sexual behaviors in PHIV^+^ youths with variable findings [5–14] and evaluation of high risk behaviors among PHIV^+^ adolescents compared to HIV-uninfected adolescents continues to be an active area of investigation. Studies delineating sexual behaviors and sexual knowledge among PHIV^+^ youths in comparison to their HIV-uninfected peers are needed with the goal of recommending informed tailored sex education programs for prevention of risky behaviors as well as promotion of sexual health. We report our experience with assessment of sexual knowledge and high risk behavior of PHIV^+^ adolescents compared to HIV-uninfected adolescents at a tertiary-care institution.

## 2. Materials and Methods

### 2.1. Study Design and Methodology

An IRB approved cross-sectional study was conducted in an urban, inner city, 200-bed tertiary-care children's hospital in Paterson, New Jersey, from December 1, 2009, to November 30, 2010. All consecutive PHIV^+^ adolescents evaluated at the Pediatric HIV-clinic during the study period were included. All of the enrolled PHIV^+^ patients were known to be born to HIV-positive mothers and had positive HIV nucleic acid testing during infancy and/or HIV antibody testing (positive immunoassay and Western blot) at 18–24 months of age. All consecutive adolescents evaluated at the routine adolescent clinic during the study period with a negative parental history for HIV and negative results of HIV antibody testing at enrollment were included as HIV-uninfected controls.

Adolescence was defined in accordance with the WHO definition [15]. Adolescents with physician documented developmental delay or with severe cognitive impairment, autism, and other pervasive developmental disorders that precluded understanding of the study questions were excluded.

Centers for Disease Control and Prevention (CDC) Youth Risk behavior Surveillance questionnaire [2] was modified to only include questions to evaluate sociodemographic information, sexual behavior, and knowledge. Details of the questionnaire are presented in Appendix. Other pieces of information for PHIV^+^ included adherence to the prescribed ART regimen over the past 7 days (7-day recall as previously described) [12]. The written questionnaires were administered to PHIV^+^ and HIV-uninfected adolescents after obtaining informed consent from the legal guardians and assent from participants. Youths over age of 18 years were allowed to consent for themselves. Questionnaires were administered to the adolescents in caregivers' absence. In addition, medical chart review of the PHIV^+^ patients was done to determine if they had a detectable viral load during the past 3 months.

### 2.2. Statistical Analysis

Comparisons between study groups for categorical variables were performed with chi-square (*χ* ^2^) or Fisher's exact test. Continuous variables were compared by *t*-tests or nonparametric tests (Mann Whitney *U* test) where appropriate and data were presented as median with IQR or means and standard deviations. SPSS version 19 (IBM, Armonk, New York) and StatXact-8 (Cytel, Cambridge, Massachusetts) were used to analyze the data. A *p* value of <0.05 was considered statistically significant and all tests were two-tailed.

## 3. Results

A total of 127 participants (27 PHIV^+^ and 100 HIV-uninfected adolescents) were enrolled (1 : 3).

### 3.1. Sociodemographic Characteristics

Both groups were similar in terms of demographics and social characteristics (Table 1). The majority of adolescents in both groups reported an annual household income of less than $20,000 (68% (18/27) PHIV^+^ and 69% (69/100) HIV-uninfected adolescents, resp., *p* = 0.81). Among the PHIV^+^ patients, all reported adherence to the prescribed antiretroviral therapy and only 1 patient had a detectable viral load (400 copies/mL).

### 3.2. Sexual Behaviors

Fifty-nine percent (16/27) of PHIV^+^ and 52% (52/100) of HIV-uninfected adolescents were sexually active (*p* = 0.52). The age at initiation of sexual activity was similar for both groups (median of 14 years (IQR 13–16 years) for PHIV^+^ and median of 14 years (IQR 12–15) for HIV-uninfected adolescents, *p* = 0.11).

Seventy-five percent (12/16) of the sexually active PHIV^+^ adolescents reported unprotected sex compared to 13% (7/52) of HIV-uninfected adolescents (*p* < 0.0001). A significantly higher proportion of PHIV^+^ adolescents had multiple (>4) sexual partners than HIV-uninfected adolescents (62% (10/16) versus 31% (16/52), *p* = 0.037). Both groups were comparable for oral sex (81% (13/16) and 71% (37/52), *p* = 0.5). A significantly greater number of PHIV^+^ males compared to HIV-uninfected males reported receptive anal intercourse (60% (9/15 males) versus 13% (7/54 males), *p* < 0.001). A significantly higher proportion of PHIV^+^ girls than HIV-uninfected adolescents reported ≥1 pregnancies during lifetime (58% (7/12) versus 13% (6/46), *p* = 0.0026). A comparable number in both groups reported regular (≥1 time per week) alcohol use (11% (3/27) PHIV^+^ versus 4% (4/100) non-HIV, *p* = 0.16) and marijuana use (7% (2/27) versus 3% (3/100), *p* = 0.28). A hundred percent of adolescents in both groups expressed their desire to have children. Only 25% (4/16) of the sexually active PHIV^+^ patients reported disclosing their seropositive status to their first sexual partners.

### 3.3. Sexual Knowledge

The median age of disclosure of positive HIV status to the PHIV^+^ group was 11 years (IQR 9–13). As many as 33% (9/27) of PHIV^+^ and 32% (32/100) of HIV-uninfected group did not know that abstinence from sex can prevent HIV transmission (*p* > 0.999). Knowledge regarding condoms as a means of HIV transmission was reported by 18% (5/27) of PHIV^+^ youths compared to 40% (40/100) of HIV-uninfected youths (*p* = 0.0043); 37% (10/27) of PHIV^+^ and 34% (34/100) of HIV-uninfected group thought that oral contraceptive pills (OCPs) alone can prevent HIV transmission (*p* = 0.82). The majority of adolescents in both groups (85% (23/27) PHIV^+^ and 90% (90/100) HIV-uninfected adolescents, *p* = 0.49) did not consider multiple sexual partners a risk factor for HIV transmission. Only 19% (5/27) of PHIV^+^ and 11% (11/100) of HIV-uninfected youths recognized anal intercourse as a means of HIV transmission (*p* = 0.32).

The sources of sex education for both groups are shown in Table 2. Majority of adolescents in both groups reported school and healthcare workers (HCW) to be the major and most useful sources of information. A significantly higher proportion of PHIV^+^ youths reported receiving sex education from HCW and found the information useful (Table 2).

## 4. Discussion

This is one of the few studies comparing sexual behaviors and knowledge among PHIV^+^ and HIV-uninfected youths. Our study showed that high risk sexual behaviors were more prevalent among PHIV^+^ adolescents; however, both groups demonstrated lack of sexual knowledge regardless of their HIV status.

The mean age of the PHIV^+^ adolescents in our study was 17 years and more than half of the PHIV^+^ adolescents were sexually active. In a previous study by Tassiopoulos et al., the mean age of PHIV^+^ cohort was 13.5 years and overall 28% were sexually active; however, among the PHIV^+^ youths aged 16 and 18 years, 53% and 67%, respectively, were noted to be sexually active [5]. This observation, similar to our study, reflects that age is an important predictor of sexual activity. In our study, as many as 75% of the sexually active PHIV^+^ adolescents reported unprotected sex. Previous studies have also noted high rates of unprotected sex among the sexually active PHIV^+^ youths (62% and 65%, resp.) [5, 12]. The median age of initiation of sexual activity among PHIV^+^ youths in our study was comparable to previous reports [5, 12].

In our study, only 25% of the sexually active PHIV^+^ patients reported disclosing their seropositive status to their first sexual partners which is lower than previously described [5]. As previously shown, nondisclosure to sexual partners is a major risk factor for transmitting HIV as well as drug-resistant viral strains, especially if the youths engage in unprotected sex [5]. We noted a high rate of pregnancy among the PHIV^+^ girls in our study, similar to previously reported data [7]. In contrast to previous studies [11, 12] we did not find a high prevalence of substance abuse among either group. Both groups of adolescents showed lack of knowledge about risk factors for HIV transmission and prevention of HIV.

Our study results reflect an overall inability to recognize the relationship between risky sexual behaviors and preventive measures, emphasizing the need to design targeted education and prevention programs for both groups. Our results also show that school and healthcare workers (HCW) stand to make the best impact in reproductive health interventions and highlight the potential pivotal role that HCW can play in providing the correct and needed information to enable adolescents to shape their choices. Empowering adolescents with useful, relevant, and developmentally appropriate information will potentially positively impact current trends in HIV transmission and promote adolescent sexual and reproductive health.

One limitation of our study was that the participants were recruited from HIV-care clinic and adolescent medical clinic, which may not be reflective of the segment of urban youths that are not followed in regular HIV care. Nonetheless, the present study adds to the evidence favoring the need for effective adolescent reproductive health interventions.

## 5. Conclusions

Our results showed a high prevalence of risky sexual behaviors among PHIV^+^ adolescents and considerable gaps in sexual knowledge among both PHIV^+^ and HIV-uninfected adolescents. These data underscore the need for targeted education and focused efforts for facilitating safe sexual practices and promoting reproductive health among adolescents.

## Figures and Tables

**Table 1:** Demographics and social characteristics of 127 perinatally HIV-infected (PHIV^+^) and HIV-uninfected adolescents (non-HIV).

	PHIV^+^ (*n* = 27)		Non-HIV (*n* = 100)		*p* value
	*N*	%	*N*	%	
*Demographics*					
Male gender	15	56	54	54	>0.999
Hispanic	10	37	34	34	0.82
African American	14	52	50	50	>0.999
Others^a^	3	11	16	16	0.76
Age, years (mean ± SD)	17 (1.7)		16 (2.9)		0.22
*Social characteristics*					
Currently enrolled in school	20	74	70	70	0.81
Arrested by police at least once	3	11	11	11	>0.999
Supported by family	24	89	91	91	0.71
At least 1 parent as caregiver	14	52	72	72	0.063
Best friends sexually active?	13	48	41	41	0.51
Alcohol use by best friends?	9	33	52	52	0.12

^a^ The other race/ethnicity includes white, Caribbean-American, and mixed race/ethnicity.

**Table 2:** Sources of sexual health education for perinatally HIV-infected (PHIV^+^) and HIV-uninfected adolescents (non-HIV).

	PHIV^+^ (*n* = 27)		Non-HIV (*n* = 100)		*p* value
	*N*	%	*N*	%	
*Source of sex education*					
School	21	77	75	75	>0.999
Healthcare workers (HCW)	18	67	31	31	**0.0015**
Media	6	22	19	19	0.78
Parents/relatives	9	33	20	20	0.19
Friends	4	15	22	22	0.59
*Source of sex education reported to be useful*					
School	14	52	53	53	>0.999
HCW	13	48	24	24	**0.018**
Media	2	7	5	5	0.63
Parents/relatives	7	26	18	18	0.41
Friends	3	11	17	17	0.56

## Appendix

### Modified CDC Youth Risk Behavior Surveillance Questionnaire [2] Administered to the Study Participants

*Components of the Questionnaire*

- Social and demographic characteristics:
  - age, gender, race/ethnicity (black, white/other/unknown, Hispanic),
  - whether supported by family (yes/no); having at least 1 parent as the caregiver (yes/no),
  - annual family income (<$20,000, >$20,000–40,000, >$40,000),
  - if enrolled in school (yes/no); if arrested by police at least once (yes/no),
  - best friends' alcohol use (yes/no); best friends' sexual activity (yes/no).
- Sexual behavior and practices:
  - sexual activity (defined as penetrative sex (vaginal or anal or both)) (yes/no),
  - age of initiation of sexual activity,
  - unprotected penetrative sex (one or more occasions of penetrative sex without a condom) (yes/no),
  - number of lifetime sexual partners (1, 2, 3, 4, >4),
  - oral sex (yes/no),
  - procreational intent (yes/no),
  - ≥1 pregnancies during lifetime (for females) (yes/no),
  - regular (≥1 time per week) alcohol use (yes/no); regular (≥1 time per week) marijuana use (yes/no).
- Sexual knowledge:
  - acquisition/transmission of HIV (knowing if HIV can be transmitted by injection drug use, vaginal sex, anal sex, mother to child) (yes/no),
  - prevention of HIV (knowing if HIV can be prevented by sexual abstinence, condom use, oral contraceptives) (yes/no),
  - risk factors for HIV (knowing that multiple partners are a risk factor for HIV) (yes/no),
  - major source of sexual health education (school, media, parents/relatives, friends, and healthcare workers); what sources of sex education were found to be useful.
- Other information for the PHIV infected group:
  - age of disclosure of HIV status,
  - seropositive status disclosure to their first sexual partner (yes/no).

## Abbreviations

WHO: World Health organization
HIV: Human Immunodeficiency Virus
PHIV: Perinatally Acquired Human Immunodeficiency Virus
PHIV^+^: Perinatally Acquired Human Immunodeficiency Virus-infected
HCW: Healthcare workers
CDC: Centers for disease control and prevention.
